# Decreased Store Operated Ca^2+^ Entry in Dendritic Cells Isolated from Mice Expressing PKB/SGK-Resistant GSK3

**DOI:** 10.1371/journal.pone.0088637

**Published:** 2014-02-11

**Authors:** Evi Schmid, Jing Yan, Meerim K. Nurbaeva, Antonella Russo, Wenting Yang, Caterina Faggio, Ekaterina Shumilina, Florian Lang

**Affiliations:** 1 Department of Physiology, University of Tübingen, Tübingen, Germany; 2 Department of Biological and Environmental Sciences, University of Messina, S.Agata-Messina, Italy; National Cancer Institute, United States of America

## Abstract

Dendritic cells (DCs), key players of immunity, are regulated by glycogen synthase kinase GSK3. GSK3 activity is suppressed by PKB/Akt and SGK isoforms, which are in turn stimulated by the PI3K pathway. Exposure to bacterial lipopolysaccharides increases cytosolic Ca^2+^-concentration ([Ca^2+^]_i_), an effect augmented in DCs isolated from mutant mice expressing PKB/SGK-resistant GSK3α,β (*gsk3^KI^*). Factors affecting [Ca^2+^]_i_ include Ca^2+^-release from intracellular stores (CRIS), store-operated Ca^2+^-entry (SOCE) through STIM1/STIM2-regulated Orai1, K^+^-dependent Na^+^/Ca^2+^-exchangers (NCKX), K^+^-independent Na^+^/Ca^2+^-exchangers (NCX) and calbindin-D28k. The present study explored whether PKB/SGK-dependent GSK3α, β-activity impacts on CRIS, SOCE, NCKX, NCX or calbindin. DCs were isolated from *gsk3^KI^* mice and respective wild-type mice (*gsk3^WT^*), [Ca^2+^]_i_ estimated from Fura2 fluorescence, Orai1, STIM1, STIM2 as well as calbindin-D28k protein abundance determined by Western blotting and mRNA levels quantified by real time PCR. As a result, thapsigargin-induced CRIS and SOCE were significantly blunted by GSK3-inhibitors SB216763 (1–10 µM, 30 min) or GSK-XIII (10 µM, 30 min) but were significantly lower in *gsk3^WT^* than in *gsk3^KI^*DCs. Orai1, STIM1 and STIM2 protein abundance was significantly lower and calbindin-D28k abundance significantly higher in *gsk3^KI^* than in *gsk3^WT^*DCs. Activity of NCKX and NCX was significantly higher in *gsk3^KI^* than in *gsk3^WT^*DCs and was significantly increased by SB216763 (1 µM, 30 min) or GSK-XIII (10 µM, 30 min). Treatment of *gsk3^WT^* DCs with SB216763 (1 µM, 4–24 h) or GSK-XIII (10 µM, 4–24 h) did not significantly modify the protein abundance of Orai1, STIM1 and STIM2. The present observations point to a dual role of GSK3 in the regulation of Ca^2+^ in DCs. Acute inhibition of GSK3 blunted the increase of [Ca^2+^]_i_ following CRIS and SOCE and stimulated NCKX/NCX activity. However, expression of PKB/SGK-resistant GSK3α, β downregulated the increase of [Ca^2+^]_i_ following CRIS and SOCE, an effect at least partially due to downregulation of Orai1, STIM1 and STIM2 expression as well as upregulation of Na^+^/Ca^2+^-exchanger activity and calbindin D28k expression.

## Introduction

The function of dendritic cells (DCs), antigen-presenting cells critically important for primary immune responses and establishment of immunological memory [1], [2], is regulated by cytosolic Ca^2+^ concentration [3]. Increase of cytosolic Ca^2+^ concentration may be accomplished in part by store operated Ca^2+^ entry (SOCE) [4]–[7], which in DCs involves the pore forming channel proteins Orai1 or Orai2 and their regulators STIM1 or STIM2 [8], [9]. The increase of cytosolic Ca^2+^ concentration is further shaped by cytosolic Ca^2+^ extrusion mechanisms such as K^+^-independent (NCX) and K^+^-dependent (NCKX) Na^+^/Ca^2+^ exchangers [10], [11]. Moreover, at least in theory, alterations of free cytosolic Ca^2+^ concentration could be blunted by cytosolic Ca^2+^ binding proteins, such as calbindin-D28k [12].

Mechanisms involved in the regulation of cytosolic Ca^2+^ activity include glycogen synthase kinase 3 (GSK3α,β) [13]. Pharmacological inhibition of GSK3 by SB216763 (3-[2,4-Dichlorophenyl]-4-[1-methyl-1H-indol-3-yl]-1H-pyrrole-2,5-dione, 10 µM, 30 min) blunts lipopolysaccharide (LPS)-induced increase of cytosolic Ca^2+^ concentration [13]. SB216763 inhibits the activating phosphorylation site tyrosine 216 of GSK3 [14]. GSK3 is further inhibited by the aminopyrazole moiety of GSK-XIII, which blocks the ATP-binding site of GSK3 [15], [16].

GSK3 participates in the regulation of DC function [17]–[21], GSK3 is active in immature DCs and suppresses DCs maturation [20]. Upon toll-like receptor activation, GSK3 fosters the development of a proinflammatory phenotype [17]–[20] and GSK3 inhibitors may suppress immune responses [19]. GSK3 thus modifies the balance between pro- and anti-inflammatory cytokine production [17], [18], [22]–[30]. Beyond that GSK3 may counteract DC survival [31]. In other cell types, GSK3 (GSK3α,β) participates in the regulation of metabolism, cell proliferation, cell differentiation and cell survival [32]–[34] and deranged GSK3β activity contributes to the pathophysiology of diabetes, cancer, inflammation, mood disorders and neurodegeneration [35]–[37].

GSK3 activity could be suppressed by phosphorylation [38], [39]. GSK3 is phosphorylated by protein kinase B (PKB/Akt) [40], [41] and the serum and glucocorticoid inducible kinase SGK1 [42], [43], which are both activated through the phosphoinositide 3 (PI3) kinase pathway [44], [45]. Replacement of the respective PKB/SGK phosphorylation sites in GSK3α and GSK3β by alanine (GSK3α^21A/21A^, GSK3β^9A/9A^) renders GSK3 activity insensitive to inhibition by PKB/SGK [46]. Gene targeted mice carrying the respective mutations (*gsk3^KI^*) are resistant to the effect of insulin on muscle glycogen synthase [46]. Moreover, in those mice alterations of renal function [47], [48], gastric acid secretion [49], catecholamine release [50], behaviour [51] and IL-10 production following IFN-β treatment [17] have been observed.

## Materials and Methods

### Ethics Statement

All animal experiments were conducted according to the recommendations of the Guide for Care and Use of Laboratory Animals of the National Institutes of Health as well as the German law for welfare of animals, and the surgical procedures on the mice were reviewed and approved by the respective government authority of the state Baden-Württemberg (Regierungspräsidium) prior to the start of the study.

### Mice

Mice were generated in which the codon of the GSK3β gene encoding Ser9 was changed to encode nonphosphorylatable alanine (GSK3β^9A/9A^), and simultaneously the codon encoding Ser21 of GSK3α was changed to encode alanine (GSK3α^21A/21A^) thus yielding the GSK3α/β^21A/21A/9A/9A^ double knockin mouse (*gsk3^KI^*) as described previously [46]. The mice were compared to corresponding wild type mice (*gsk3^WT^*).

### Cell Culture

Dendritic cells (DCs) were cultured from bone marrow of 7–12 week old mice. Bone marrow derived cells were flushed out of the cavities from the femur and tibia with PBS. Cells were then washed twice with RPMI and seeded out at a density of 2×10^6^ cells/10 ml per 60-mm dish. Cells were cultured for 8 days in RPMI 1640 (GIBCO, Carlsbad) containing: 10% FCS, 1% penicillin/streptomycin, 1% glutamine, 1% non-essential amino acids (NEAA) and 0.05% β-mercaptoethanol. Cultures were supplemented with GM-CSF (35 ng/mL, Immunotools) and fed with fresh medium containing GM-CSF on days 3 and 6. Experiments were performed on DCs at days 7–9.

### Western Blotting

The protein expression levels were analyzed by Western blotting. In brief, DCs from *gsk3^KI^* or *gsk3^WT^* mice were washed with ice cold phosphate-buffered saline (PBS) and cells were lysed with cell lysis buffer (Cell Signaling Technology, Inc., New England Biolabs). The extracts were centrifuged at 13,000 rpm for 20 min at 4°C and the protein concentration of the supernatant was determined. Total protein (30 µg) was subjected to 10% SDS-PAGE. Proteins were transferred to a nitrocellulose membrane (VWR) and the membranes were then blocked for 1 h at room temperature with 10% non- fat dried milk in tris-buffered saline (TBS) containing 0.1% Tween-20. For immunoblotting the membranes were incubated overnight at 4 °C with antibodies directed against GSK3α/β (D75D3, XP™ antibody, 1∶1000, Cell Signaling Technology, Inc., New England Biolabs, 46, 51 kDa), phospho-GSK3α/β (Ser21/9, 1∶1000, Cell Signaling Technology, Inc., New England Biolabs, 46, 51 kDa), Orai1 (1∶500, Proteintech, Manchester), STIM1 (1∶300, Cell Signaling Technology, Inc., New England Biolabs), STIM2 (1∶300 Cell Signaling Technology, Inc., New England Biolabs) or calbindin-D28k (1∶200, SWANT, Switzerland). A GAPDH antibody (1∶1000, Cell Signaling Technology, Inc., New England Biolabs) was used for a loading control. Specific protein bands were visualized after subsequent incubation with a 1∶3000 dilution of anti-rabbit IgG conjugated to horseradish peroxidase and a Super Signal Chemiluminescence detection procedure (GE Healthcare, UK). Specific bands were quantified by Quantity one software (Bio rad gel doc system, Chemidoc XRS). Levels of each protein were expressed as the ratio of signal intensity for the target protein relative to that of GAPDH.

### Real-time PCR

Total RNA was extracted from mouse dendritic cells in TriFast (Peqlab, Erlangen, Germany) according to the manufacturer's instructions. After DNAse digestion reverse transcription of total RNA was performed using Transcriptor High Fidelity cDNA Synthesis kit (Roche). Polymerase chain reaction (PCR) amplification of the respective genes were set up in a total volume of 20 µl using 40 ng of cDNA, 500 nM forward and reverse primer and 2x GoTaq® qPCR Master Mix SYBR Green (Promega Corporation, Madison, WI, USA) according to the manufacturer's protocol. Cycling conditions were as follows: initial denaturation at 95°C for 2 min, followed by 40 cycles of 95°C for 15 sec, 55°C for 15 sec and 72°C for 20 sec. For the amplification the following primers were used (5′->3′orientation):

Orai1, fw CATGGTAGCGATGGTGGAAGTC rev TGCTfCATCGTCTTTAGTGCCT;

Orai2, fw ATGGTGGCCATGGTGGAGGT rev ATTGCCTTCAGCGCCTGCA;

STIM1, fw CTTGGCCTGGGATCTCAGAG rev TCAGCCATTGCCTTCTTGCC;

STIM2, fw GCAGGATCTTTAGCCAGAAG rev ACATCTGCTGTCACGGGTGA;

Calbindin, fw TCCCTCACCTAGAGATAGAAGCAGCGCAG

rev AGACAGCAGAATCGAGGAGTCTGCTGCTC;

Tbp, fw CAAGCTGGAGGTGATCATCG rev TCCACAGTGCTCTTGAATTCG.

Specificity of PCR products was confirmed by analysis of a melting curve. Real-time PCR amplifications were performed on a CFX96 Real-Time System (Bio-Rad). All experiments were done in duplicate. Amplification of the house-keeping gene Tbp (TATA binding protein) was performed to standardize the amount of sample RNA. Relative quantification of gene expression was achieved using the Δct method as described earlier [52].

### Measurement of intracellular Ca^2+^

To determine cytosolic Ca^2+^ concentration, the cells were loaded with Fura-2/AM (2 µM, Molecular Probes, Goettingen, Germany) for 15 min at 37°C. Fluorescence measurements were carried out with an inverted phase-contrast microscope (Axiovert 100, Zeiss, Oberkochen, Germany). Cells were excited alternatively at 340 or 380 nm and the light was deflected by a dichroic mirror into either the objective (Fluar 40×/1.30 oil, Zeiss, Oberkochen, Germany) or a camera (Proxitronic, Bensheim, Germany). Emitted fluorescence intensity was recorded at 505 nm and data acquisition was accomplished by using specialized computer software (Metafluor, Universal Imaging Downingtown, USA). The corresponding ratios (F_340_/F_380_) were used to obtain intracellular Ca^2+^ concentrations. The following equation was used: [Ca^2+^]_free_  =  K_d_ x ((R-R_min_)/(R_max_-R)) x S_f_ (K_d_  =  dissociation constant of Fura-2; R  =  ratio of emission intensity, exciting at 340 nm, to emission intensity, exciting at 380 nm; R_min_  =  ratio at zero free Ca^2+^; R_max_  =  ratio at saturating Ca^2+^; S_f_  =  instrumental constant). As a measure for the increase of cytosolic Ca^2+^ concentration, the slope and peak of the changes in intracellular Ca^2+^ concentration were determined in each experiment.

To measure SOCE, changes in cytosolic Ca^2+^ were monitored upon depletion of the intracellular Ca^2+^ stores. Experiments were carried out prior to and during exposure of the cells to the Ca^2+^-free solution (see below). In the absence of Ca^2+^, the intracellular Ca^2+^ stores were depleted by inhibition of the vesicular Ca^2+^ pump by thapsigargin (1 µM; Molecular Probes). Readdition of Ca^2+^ allowed assessing the SOCE. To monitor the activity of K^+^ dependent (NCKX) or K^+^ independent (NCX) Na^+^/Ca^2+^ exchangers, changes in cytosolic Ca^2+^ were determined upon replacement of extracellular Na^+^ by *N*-methyl-D-glucamine (NMDG) in Ringer solution for NCX or NCKX. In some experiments DCs were treated with GSK3 inhibitors SB2167633 ([2,4-Dichlorophenyl]-4-[1-methyl-1H-indol-3-yl]-1H-pyrrole-2,5-dione, 100 nM - 10 µM, Enzo Life Sciences, Lausen, Switzerland) or GSK3 inhibitor XIII (GSK-XIII, 10 µM, company) [16] for 30 min before the experiment (15 min before addition of Fura-2A/M and then 15 min together with Fura-2A/M).

The Ringer solution contained (in mM): 125 NaCl, 5 KCl, 1.2 MgSO_4_, 32.2 Hepes, 2 Na_2_HPO_4_, 2 CaCl_2_, and 5 glucose at pH 7.4 (NaOH). Ca^2+^-free solution contained (in mM): 125 NaCl, 5 KCl, 1.2 MgSO_4_, 2 Na_2_HPO_4_, 32.2 Hepes, 0.5 EGTA, and 5 glucose at pH 7.4 (NaOH). Ringer solution for NCX contained (in mM): 130 NaCl, 2 CaCl_2_, 2 MgCl_2_, 10 HEPES, and 10 glucose at pH 7.4 (NaOH). Ringer solution for NCKX contained (in mM): 90 NaCl, 40 KCl, 2 CaCl_2_, 2 MgCl_2_, 10 HEPES, and 10 glucose at pH 7.4 (NaOH). The Na^+^-free solutions were identical, except that NaCl was replaced by 130 or 90 mM NMDG to measure NCX or NCKX, respectively.

### Statistics

Data are provided as means ± SEM, *n* represents the number of independent experiments. All data were tested for significance using unpaired Student *t*-test, Mann–Whitney U test or ANOVA (Kruskal-Wallis Test, Dunnett test). Only results with p<0.05 were considered statistically significant.

## Results

**Figure 1:**
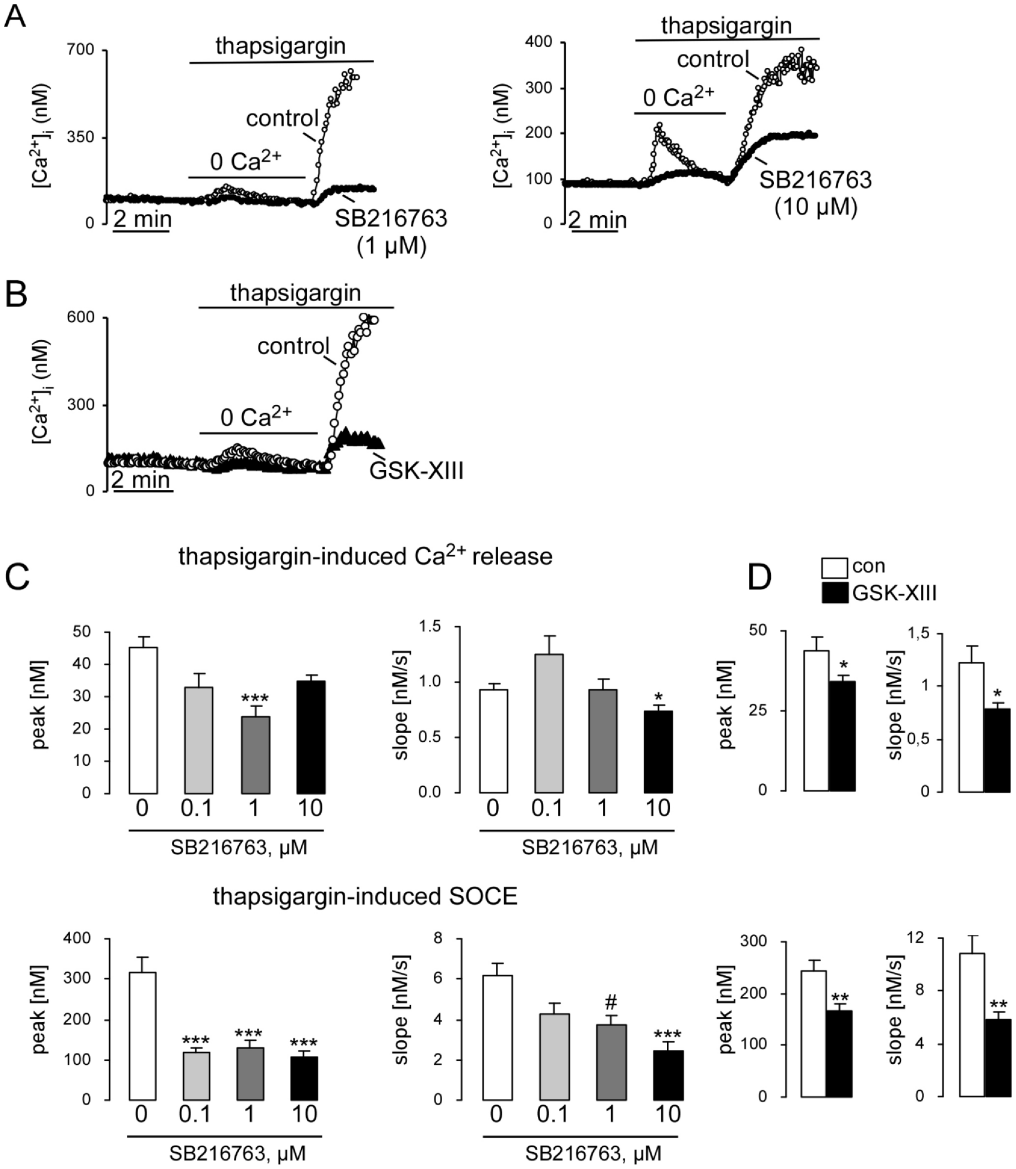
Effect of GSK3 inhibitors SB216763 or GSK-XIII on thapsigargin-induced intacellular Ca^2+^ release and subsequent SOCE in DCs. A. Representative original tracings showing intracellular Ca^2+^ concentrations ([Ca^2+^]_i_) in Fura-2/AM loaded wild type (*gsk3^WT^*) dendritic cells (DCs) prior to and following removal of extracellular Ca^2+^, addition of the sarco-endoplasmic Ca^2+^ ATPase (SERCA) inhibitor thapsigargin (1 µM) and readdition of extracellular Ca^2+^, all in the absence (open circles) and presence (closed circles) of GSK3 inhibitor SB216763 (3-[2,4-Dichlorophenyl]-4-[1-methyl-1H-indol-3-yl]-1H-pyrrole-2,5-dione, added 30 min before the experiment) 1 µM (left) or 10 µM (right). **B.** Representative original tracings showing [Ca^2+^]_i_ in Fura-2/AM loaded *gsk3^WT^* without (open circles) and with (closed triangles) presence of GSK3 inhibitor GSK-XIII (10 µM, 30 min) DCs prior to and following removal of extracellular Ca^2+^, addition of SERCA inhibitor thapsigargin (1 µM) and readdition of extracellular Ca^2+^. **C.** Arithmetic means ± SEM (n = 16–83) of the peak (left) and slope (right) values of [Ca^2+^]_i_ increase following addition of thapsigargin reflecting Ca^2+^ release from intracellular stores (upper bars) and of [Ca^2+^]_i_ increase following readdition of extracellular Ca^2+^ reflecting store operated Ca^2+^ entry (SOCE, lower bars) in *gsk3^WT^* DCs incubated in the presence and absence of GSK3 inhibitor SB216763 (100 nM, 1 µM, 10 µM, 30 min). *(p<0.05), ***(p<0.001), ANOVA Kruskal-Wallis Test, #(p<0.05), Mann–Whitney U test. **D.** Arithmetic means ± SEM (n = 41–53) of the peak (left) and slope (right) values of [Ca^2+^]_i_ increase upon Ca^2+^ release from intracellular stores (upper bars) and upon SOCE (lower bars) in *gsk3^WT^* DCs incubated in the presence and absence of GSK3 inhibitor GSK-XIII (10 µM, 30 min). *(p<0.05), **(p<0.001), unpaired *t*-test or Mann–Whitney U test.

The present study explored whether glycogen synthase kinase 3 (GSK3) modifies the increase of cytosolic Ca^2+^ concentration ([Ca^2+^]_i_) in dendritic cells (DCs) following stimulation of intracellular Ca^2+^ release by inhibition of the sarco/endoplasmic Ca^2+^ ATPase (SERCA) with thapsigargin in the absence of extracellular Ca^2+^ and/or the increase of [Ca^2+^]_i_ during store operated Ca^2+^ entry (SOCE) following readdition of extracellular Ca^2+^. The first series of experiments was performed in the presence and absence of the GSK3 inhibitor SB216763 (3-[2,4-Dichlorophenyl]-4-[1-methyl-1H-indol-3-yl]-1H-pyrrole-2,5-dione) added at different concentrations (100 nM – 10 µM) 30 min before the experiment. As illustrated in Fig. 1(A, C), treatment of DCs from wild type mice with thapsigargin in the absence of extracellular Ca^2+^ resulted in a transient increase of [Ca^2+^]_i_ reflecting intracellular Ca^2+^ release. The subsequent readdition of extracellular Ca^2+^ was followed by a rapid increase of [Ca^2+^]_i_ reflecting SOCE. Following addition of SB216763 the increase of [Ca^2+^]_i_ upon thapsigargin treatment (Ca^2+^ release) and following readdition of extracellular Ca^2+^ (SOCE) were blunted by 1 µM and 10 µM of SB216763 (Fig. 1A, C). Those results were confirmed by using another GSK3 inhibitor, GSK-XIII (5-methyl-1Hpyrazol-3-yl-2-phenylquinazolin-4-yl-amine) [16]. Pretreatment of DCs wih GSK-XIII (10 µM, 30 min) resulted in a statistically significant decrease of Ca^2+^ release and SOCE (Fig. 1B, D).

**Figure 2:**
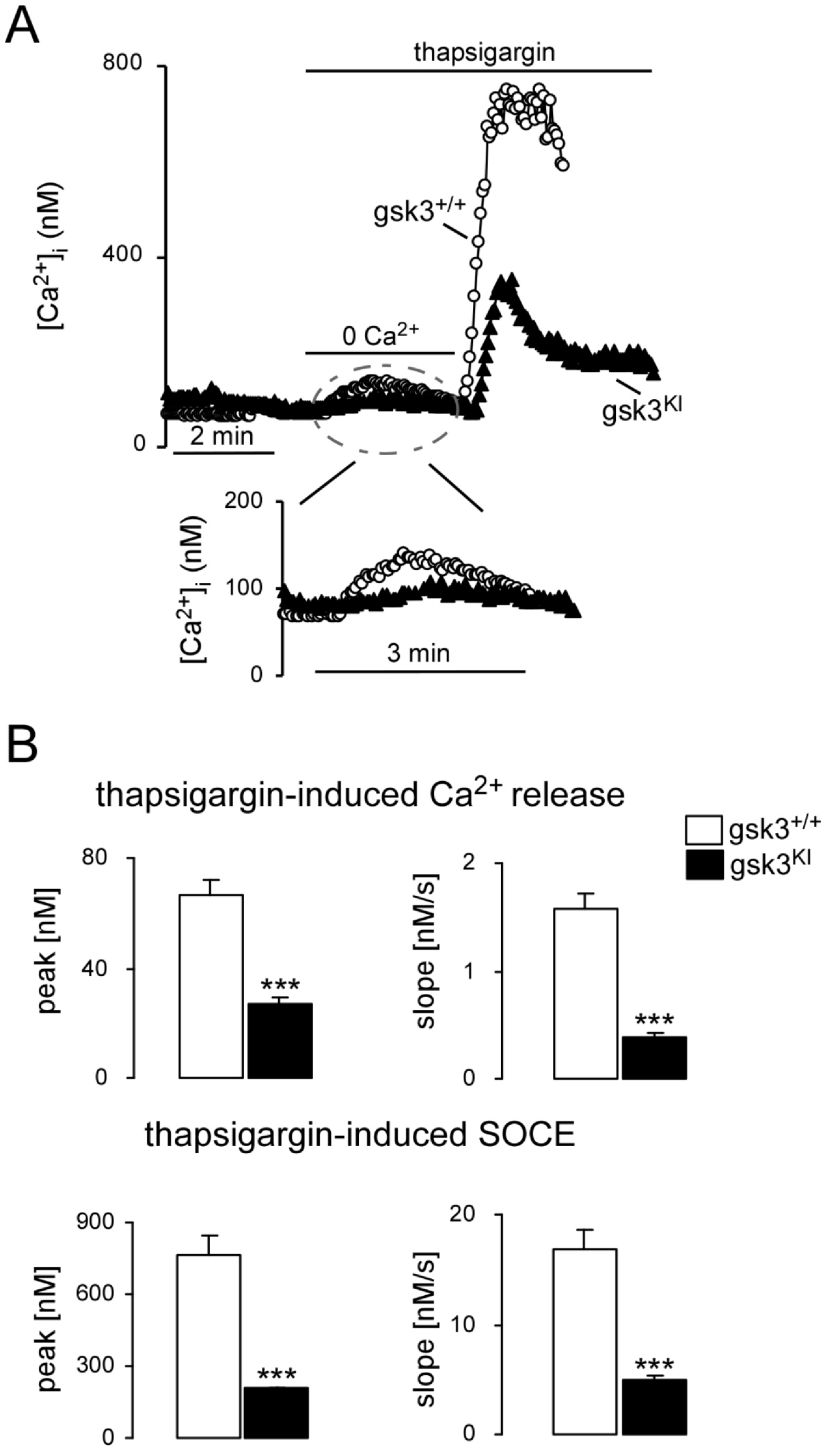
Thapsigargin-induced intacellular Ca^2+^ release and subsequent SOCE in DCs from *gsk3^KI^* and *gsk3^WT^* mice. **A.** Representative original tracings showing [Ca^2+^]_i_ in Fura-2/AM loaded *gsk3^WT^* (open circles) and *gsk3^KI^* (closed triangels) DCs prior to and following removal of extracellular Ca^2+^, addition of SERCA inhibitor thapsigargin (1 µM) and readdition of extracellular Ca^2+^. **B.** Arithmetic means ± SEM (n = 44–59) of the peak (left) and slope (right) values of [Ca^2+^]_i_ increase upon Ca^2+^ release from intracellular stores (upper bars) and upon SOCE (lower bars) in *gsk3^WT^* DCs (white bars) and *gsk3^KI^* DCs (black bars). ***(p<0.001), unpaired *t*-test.

In a second series of experiments the increase of [Ca^2+^]_i_ following intracellular Ca^2+^ release and subsequent SOCE was monitored in DCs isolated from mice expressing PKB/Akt and SGK insensitive GSK3α,β (*gsk3^KI^*) and DCs isolated from wild type mice (*gsk3^WT^*). As illustrated in Fig. 2, thapsigargin induced an increase of [Ca^2+^]_i_ due to Ca^2+^ release and readdition of Ca^2+^ triggered SOCE, effects both significantly blunted in *gsk3^KI^* DCs as compared to *gsk3^WT^* DCs.

**Figure 3:**
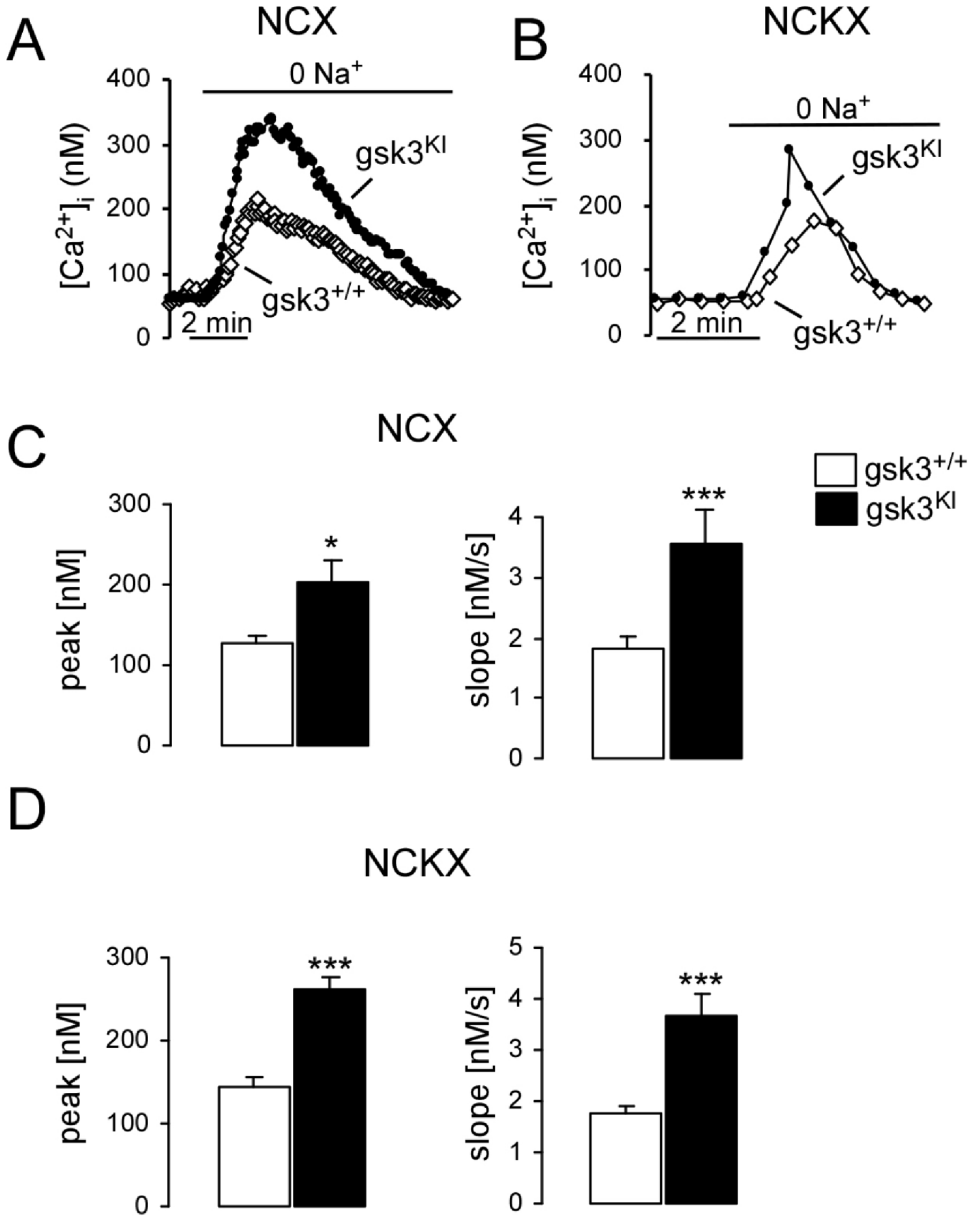
K^+^ independent (NCX) and K^+^ dependent (NCKX) Na^+^/Ca^2+^ exchanger activity in DCs from *gsk3^KI^* and *gsk3^WT^* mice. **A,B.** Representative original tracings showing [Ca^2+^]_i_ in Fura-2/AM loaded *gsk3^WT^* (open diamonds) and *gsk3^KI^* (closed circles) DCs prior to and following removal of external Na^+^ (0 Na^+^) at 0 mM K^+^ (**A**) and at 40 mM K^+^ (**B**). **C,D.** Arithmetic means ± SEM of the peak (left) and slope (right) values of [Ca^2+^]_i_ increase following removal of external Na^+^ at 0 mM K^+^ (**C**, n = 95–129) and at 40 mM K^+^ (**D**, n = 27–34) in *gsk3^WT^* DCs (white bars) and *gsk3^KI^* DCs (black bars). *(p<0.05), ***(p<0.001), unpaired *t*-test.

Further experiments were performed to elucidate underlying mechanisms. The increase of [Ca^2+^]_i_ following intracellular Ca^2+^ release and/or SOCE is expected to be blunted by stimulation of Ca^2+^ extrusion, such as activation of Na^+^/Ca^2+^ exchangers. The increase of [Ca^2+^]_i_ following removal of extracellular Na^+^ was taken as evidence for Na^+^/Ca^2+^ exchanger activity. In order to discriminate between K^+^-independent (NCX) and K^+^-dependent (NCKX) Na^+^/Ca^2+^ exchangers, experiments were performed in the absence (0 mM) or in the presence of high (40 mM) extracellular K^+^ concentrations. In the absence and in the presence of K^+^, the increase of [Ca^2+^]_i_ following removal of extracellular Na^+^ was significantly higher in *gsk3^KI^* DCs than in *gsk3^WT^* DCs (Fig. 3). Accordingly, the activity of both, NCX and NCKX was significantly higher in *gsk3^KI^* DCs than in *gsk3^WT^* DCs.

**Figure 4:**
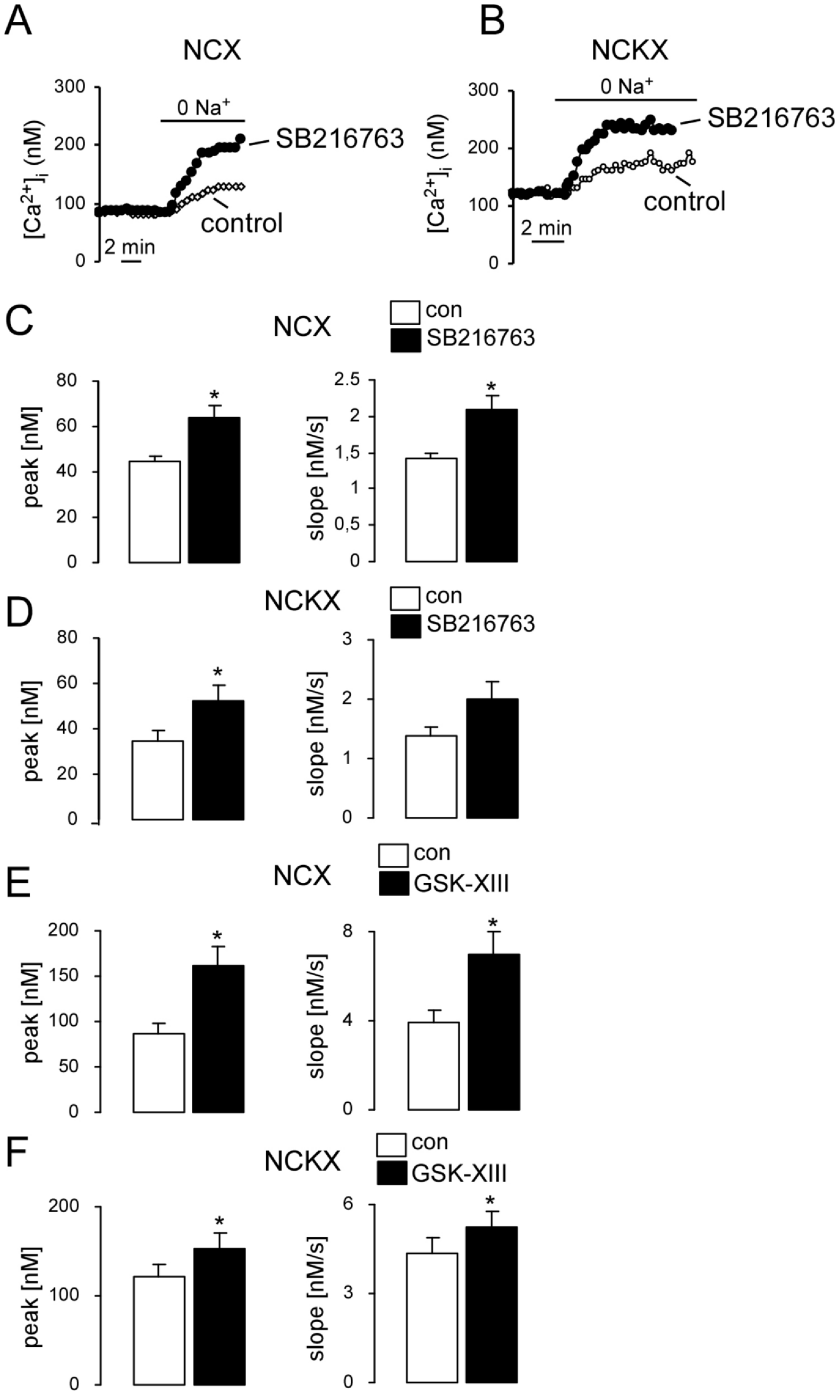
Effects of GSK3 inhibitors SB216763 or GSK-XIII on K^+^ independent (NCX) and K^+^ dependent (NCKX) Na^+^/Ca^2+^ exchanger activity in DCs. **A,B.** Representative original tracings showing [Ca^2+^]_i_ in Fura-2/AM loaded *gsk3^WT^* without (control, open circles) or with (closed circles) SB216763 treatment (1 µM, 30 min) DCs prior to and following removal of external Na^+^ (0 Na^+^) at 0 mM K^+^ (A) and at 40 mM K^+^ (B). **C,D.** Arithmetic means ± SEM of the peak (left) and slope (right) values of [Ca^2+^]_i_ increase following removal of external Na^+^ at 0 mM K^+^ (**C**, n = 24–51) and at 40 mM K^+^ (**D**, n = 43–46) in *gsk3^WT^* DCs, without (white bars) or with (black bars) SB216763 treatment (1 µM, 30 min). *(p<0.05), unpaired *t*-test. **E,F.** Arithmetic means ± SEM of the peak (left) and slope (right) values of [Ca^2+^]_i_ increase following removal of external Na^+^ at 0 mM K^+^ (**E**, n = 47–82) and at 40 mM K^+^ (**F**, n = 43–46) in *gsk3^WT^* DCs without (white bars) or with (black bars) GSK-XIII treatment (10 µM, 30 min). *(p<0.05), unpaired *t*-test or Mann–Whitney U test.

A short-term inhibition of GSK3 by either SB216763 (1 µM, 30 min, Fig. 4A–D) or GSK-XIII (10 µM, 30 min, Fig. 4E, F) also resulted in a significant upregulation of NCX and NCKX activity.

**Figure 5:**
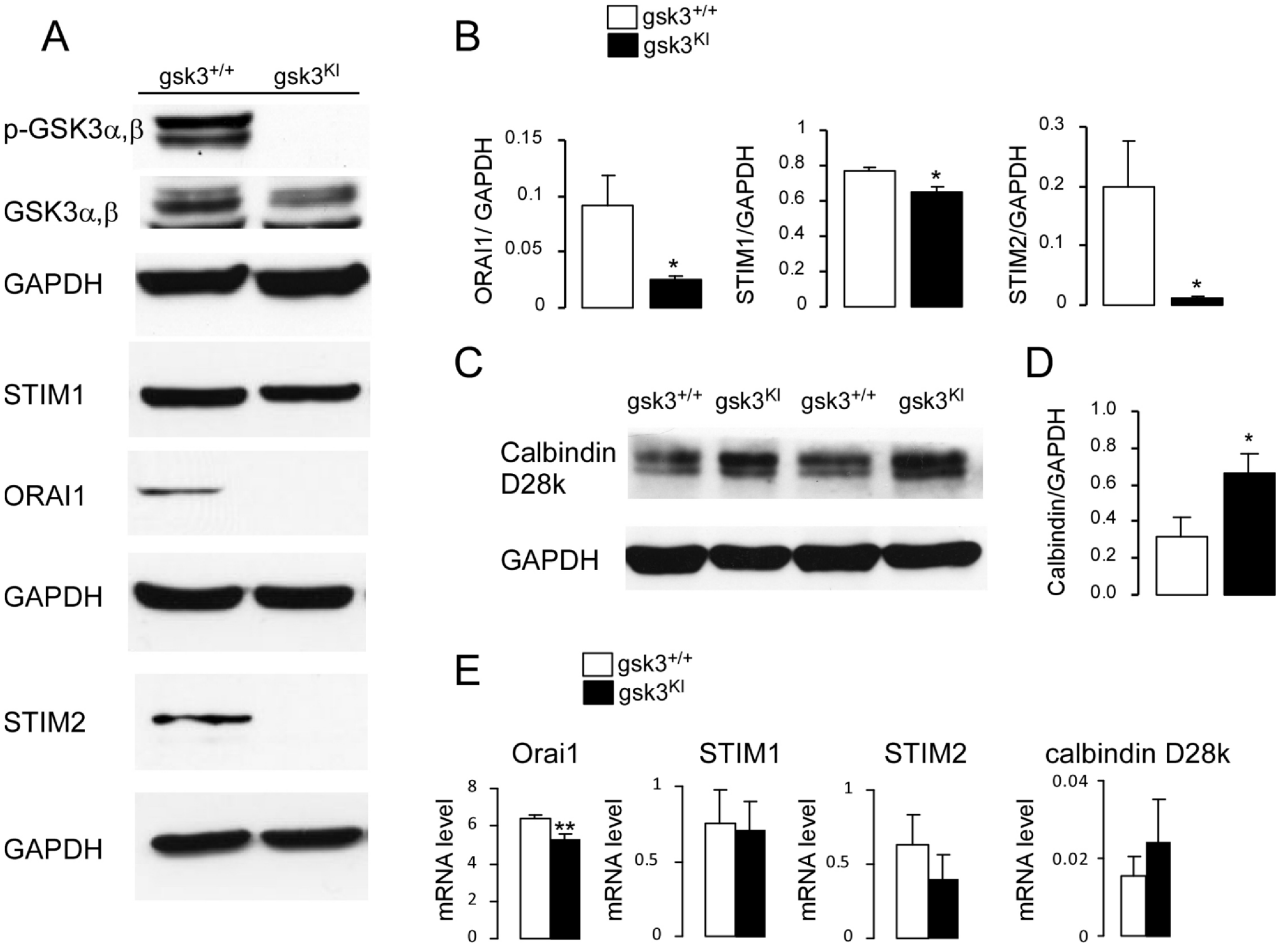
Orai1, STIM1, STIM2 and calbindin-D28k mRNA and protein abundance in DCs from *gsk3^KI^* and *gsk3^WT^* mice. **A.** Original western blot showing the protein abundance of phosphorylated (p, Ser21/9) GSK3α,β, total GSK3α,β and respective GAPDH, STIM1, Orai1 and respective GAPDH, STIM2 and respective GAPDH in DCs derived from bone marrow of *gsk3^KI^* and *gsk3^WT^* mice. Blots were stripped and reprobed with a GAPDH antibody to determine equal protein loading. Also the blot of p-GSK3 was stripped and reprobed with GSK3 antibody. **B.** Arithmetic means ± SEM (n = 4 independent experiments) of the relative (to GAPDH) protein abundance of Orai1, STIM1 and STIM2 in *gsk3^WT^* DCs (white bars) and *gsk3^KI^* DCs (black bars). *(p<0.05), unpaired *t*-test. **C.** Original western blot showing the protein abundance of calbindin-D28k (first lane) and GAPDH (2^nd^ lane), in DCs derived from bone marrow of *gsk3^KI^* and *gsk3^WT^* mice. **D.** Arithmetic means ± SEM (n = 8 independent experiments) of the relative (to GAPDH) protein abundance of calbindin-D28k in *gsk3^WT^* DCs (white bar) and *gsk3^KI^* DCs (black bar). *(p<0.05), unpaired *t*-test. **E.** Arithmetic means (± SEM, n = 5–9) of the abundance of mRNA encoding Orai1, STIM1, STIM2, and calbindin-D28k in *gsk3^WT^* DCs (white bar) and *gsk3^KI^* DCs (black bar) as assessed by real-time PCR using TBP mRNA as a reference gene. **(p<0.01), unpaired *t*-test.

Western blotting and real time PCR were employed to quantify, respectively, the protein and mRNA abundance of the pore forming Ca^2+^ release activated Ca^2+^ channel (CRAC) moiety Orai1 and its regulators STIM1 and STIM2 in *gsk3^WT^* and *gsk3^KI^* DCs. As illustrated in Fig. 5 (A, B, E), the transcript abundance of Orai1 and the protein abundance of Orai1, STIM1 and STIM2 were all significantly lower in *gsk3^KI^* DCs than in *gsk3^WT^* DCs. The transcript abundance of STIM1, and STIM2 were not significantly different between genotypes. To confirm that the mutation in *gsk3^KI^* DCs disrupted GSK3α/β phosphorylation, phosphorylated GSK3 and total GSK3 were determined in *gsk3^WT^* and *gsk3^KI^* DCs. Indeed, phosphorylated GSK3 was observed in DCs from *gsk3^WT^* mice but not in DCs from *gsk3^KI^* mice (Fig. 5A).

An increase of [Ca^2+^]_i_ could further be modified by cytosolic Ca^2+^ buffering, a function of Ca^2+^ binding proteins such as calbindin-D28k. Accordingly, Western blotting and RT-PCR were employed to quantify calbindin-D28k expression in *gsk3^WT^* and *gsk3^KI^* DCs. As illustrated in Fig. 5 (C–E), the protein, but not transcript abundance of calbindin D28k was significantly higher in *gsk3^KI^* DCs than in *gsk3^WT^* DCs.

**Figure 6:**
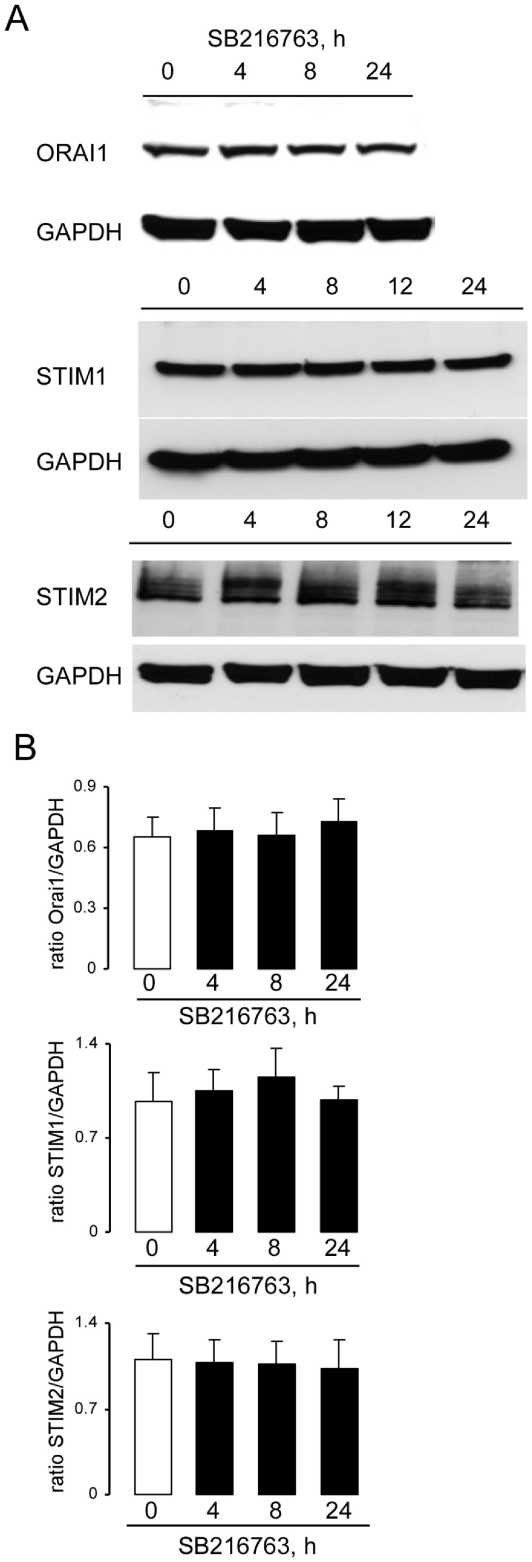
Effects of GSK3 inhibitor SB216763 on Orai1, STIM1, and STIM2 protein abundance in DCs. **A.** Original western blot showing the protein abundance of Orai1 and respective GAPDH, STIM1 and respective GAPDH, STIM2 and respective GAPDH in DCs without (control) and with SB216763 treatment (1 µM, 4–24 h). Blots were stripped and reprobed with a GAPDH antibody to determine equal protein loading. **B.** Arithmetic means ± SEM (n = 4–5 independent experiments) of the relative (to GAPDH) protein abundance of Orai1, STIM1 and STIM2 in DCs without (white bar) and with SB216763 treatment (1 µM, 4–24 h, black bars).

**Figure 7:**
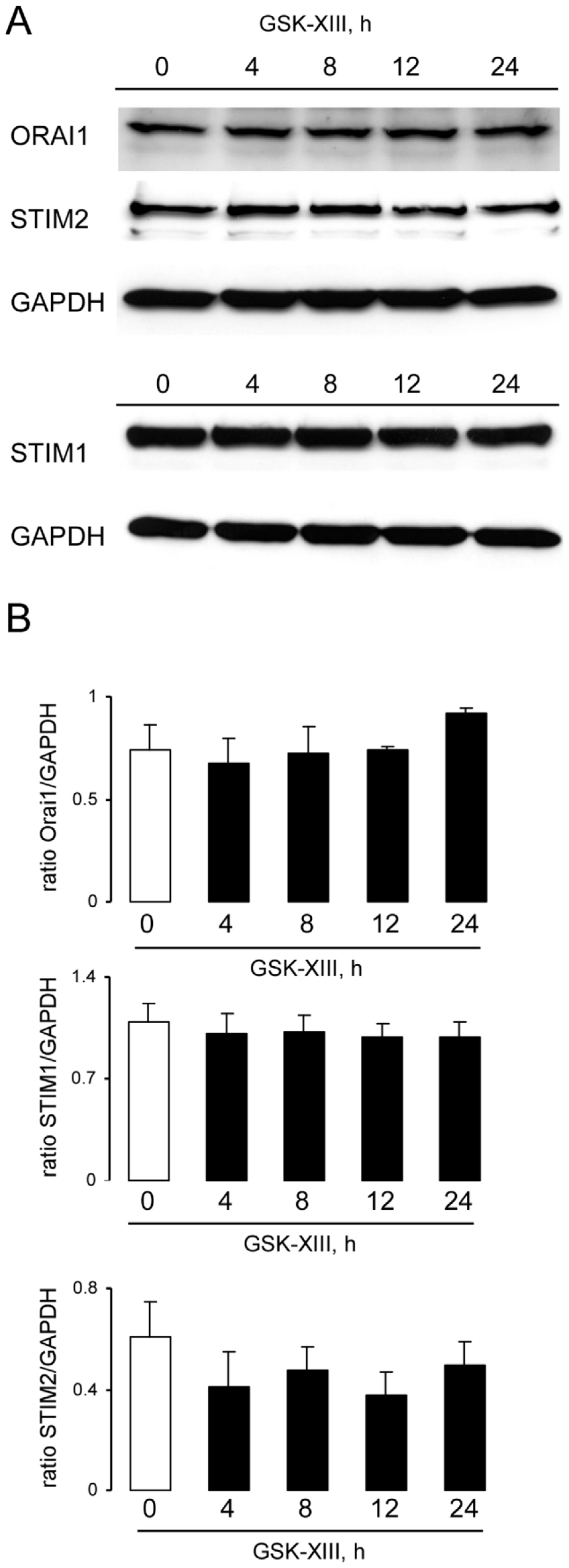
Effects of GSK3 inhibitor GSK-XIII on Orai1, STIM1, and STIM2 protein abundance in DCs. **A.** Original western blot showing the protein abundance of Orai1, STIM2 and respective GAPDH, STIM1 and respective GAPDH, in DCs without (control) and with GSK-XIII treatment (10 µM, 4–24 h). Blots were stripped and reprobed with a GAPDH antibody to determine equal protein loading. **B.** Arithmetic means ± SEM (n = 4–5 independent experiments) of the relative (to GAPDH) protein abundance of Orai1, STIM1 and STIM2 in DCs without (white bar) and with (black bars) GSK-XIII treatment (10 µM, 4–24 h).

Treating *gsk3^WT^* DCs with GSK3 inhibitors SB216763 (1 µM, 4 h–24 h, Fig. 6) or GSK-XIII (10 µM, 4 h–24 h, Fig. 7) did not significantly modify Orai1, STIM1 and STIM2 protein abundance.

## Discussion

The present observations reveal a dual role of glycogen synthase kinase 3 (GSK3) in the regulation of dendritic cell (DC) Ca^2+^ signaling. At the one hand acute inhibition of GSK3 with the GSK3 inhibitors SB216763 or GSK-XIII blunts the increase of cytosolic Ca^2+^ concentration ([Ca^2+^]_i_) following inhibition of the sarco/endoplasmic Ca^2+^ ATPase (SERCA) with thapsigargin in the absence of extracellular Ca^2+^ as well as the store operated Ca^2+^ entry (SOCE) following readdition of extracellular Ca^2+^. On the other hand, disruption of PKB/Akt and SGK dependent GSK3α,β phosphorylation downregulates both, intracellular Ca^2+^ release and SOCE. Phosphorylation of GSK3 by PKB/Akt [40], [41] and SGK [42], [43] inhibits GSK3 activity. Accordingly, disruption of the PKB/Akt and SGK dependent phosphorylation is expected to prevent the inhibition of GSK3 following stimulation of the phosphoinositide 3 (PI3) kinase pathway [46].

GSK3 is apparently active in immature DCs [20]. The kinase is transiently phosphorylated upon lipopolysaccharide (LPS) stimulation [18], [19] but still contributes to the development of a proinflammatory phenotype of DCs [20]. Pharmacological inhibition of GSK3 has previously been shown to attenuate IL-12 [18]–[20], IL-6 and TNFα [20], [53] production and to enhance IL-10 production [18], [19]. Moreover, GSK3 inhibitors blunt the increase of IL12p70 secretion from monocyte-derived DCs following PI3 kinase inhibition with wortmannin [20].

As shown earlier [13], the increase of [Ca^2+^]_i_ following treatment of DCs with bacterial LPS was virtually abolished in the presence of GSK3 inhibitor SB216763. Those observations are consistent with inhibition of SOCE by the GSK3 inhibitor. Notably, the inhibitor interferes with both, intracellular Ca^2+^ release and SOCE. Thus, GSK3 may be involved in the signaling leading to emptying of intracellular stores. Since the GSK3 inhibitor SB216763 was effective within a few minutes it apparently disrupted activation or inactivation of existing proteins. Moreover, as shown in the present study, both K^+^ dependent (NCKX) and K^+^ independent (NCX) Na^+^/Ca^2+^ exchangers are stimulated by short-term inhibition of GSK3 by either SB216763 or GSK-XIII. Enhanced extrusion of Ca^2+^ via these transporters could also underlie the reduced SOCE.

In contrast, the decreased SOCE in DCs isolated from *gsk3^KI^* mice is at least partially due to decreased expression of Orai1, STIM1 and STIM2. Moreover, the blunted increase of [Ca^2+^]_i_ in DCs from *gsk3^KI^* mice during intracellular Ca^2+^ release and SOCE are in part due to enhanced Ca^2+^ buffering due to increased expression of calbindin-D28k. The differences between DCs from *gsk3^KI^* mice and DCs from *gsk3^WT^* mice is thus partially due to altered gene expression. The blunted increase of [Ca^2+^]_i_ during intracellular Ca^2+^ release and SOCE is further in part due to enhanced activity of both K^+^ dependent (NCKX) and K^+^ independent (NCX) Na^+^/Ca^2+^ exchangers. In contrast, inhibition of GSK3 by either SB216763 or GSK-XIII did not modify expression of Orai1, STIM1 and STIM2.

At least in theory, the differences between DCs from *gsk3^KI^* mice and DCs from *gsk3^WT^* mice may be an indirect result of the altered regulation of GSK3 activity in other cell types, which in turn influence gene expression in DCs. Along those lines PKB/Akt and SGK1 resistance of GSK3 influences steroid hormone release [47], catecholamine release [50], and function of lymphocytes [54]. To the extent that those or further alterations of hormonal or cellular environment do imprint DCs *in vivo* prior to isolation, the DCs could remain altered following isolation and subsequent culture.

In conclusion, GSK3 plays a dual role in the regulation of cytosolic Ca^2+^ concentration in dendritic cells. Acutely, GSK3 activity is required for the full effect of LPS stimulation and SERCA inhibition on cytosolic Ca^2+^ activity. On the other hand, disruption of PKB/Akt and SGK dependent phoshorylation of GSK3 downregulates Orai1, STIM1 and STIM2 expression, upregulates calbindin-D28k expression and enhances the activity of K^+^ dependent (NCKX) and K^+^ independent (NCX) Na^+^/Ca^2+^ exchangers in dendritic cells.
